# Supplementary material for: Cylindracin, a Fruiting Body-Specific Protein of Cyclocybe cylindracea, Represses the Egg-Laying and Development of Caenorhabditis elegans and Drosophila melanogaster
Source: Toxins (Basel). 2025 Mar 1;17(3):118. doi: 10.3390/toxins17030118 (PMC11946224; doi:10.3390/toxins17030118)
Supplement: Supplementary file 1 [file toxins-17-00118-s001.zip › toxins-3453474-supplementary.pdf]

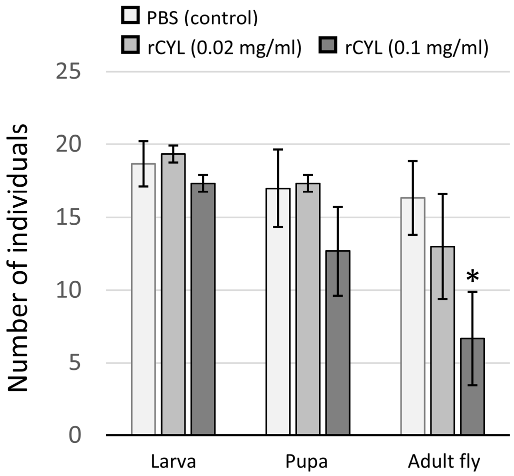

Supplemental Figure S1. Inhibitory effect of rCYL on the development of *Drosophila*.
To test the effect of rCYL on the development of *Drosophila*, 20 eggs of adult flies reared on standard medium were placed onto rCYL-medium or PBS-medium and incubated at 25°C. The total numbers of larvae, pupae, and adult flies that developed on each medium were determined after 2, 9, and 13 days, respectively. All experiments were performed in triplicate per condition, and the mean values ± SD are shown. Statistically significant differences from the control were determined using the Student's *t*-test and are indicated as $^*p < 0.05$.

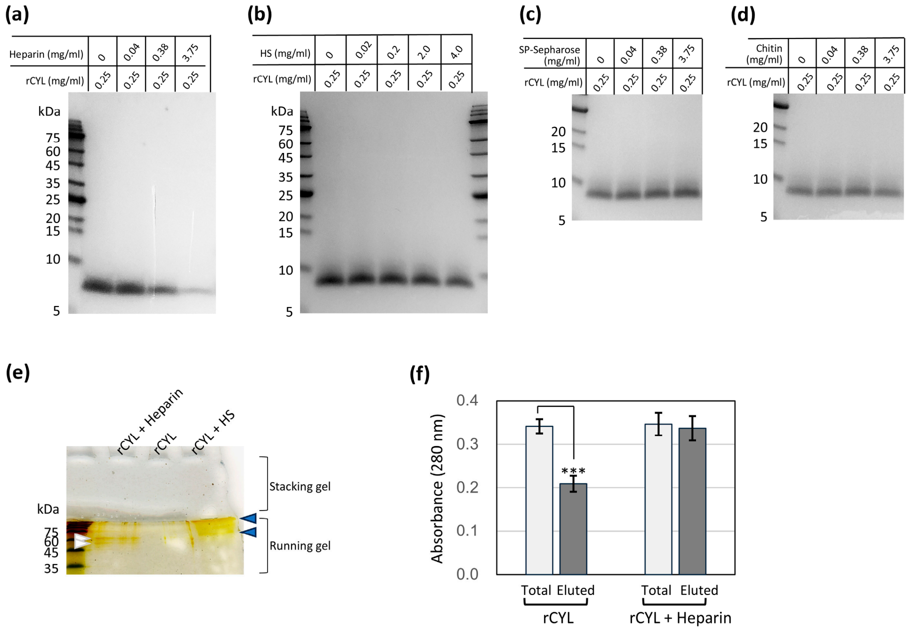

Supplemental Figure S2. Polysaccharide binding assay.
rCYL at 0.25 mg/ml was incubated with heparin (from porcine small intestine) (a), heparan sulfate (HS; from bovine kidney) (b), SP–Sepharose (c), or chitin (from crab shell) (d) at 0.02–4.0 mg/ml in 20 mM sodium phosphate, pH 7.0, and 150 mM NaCl, at 25°C for 30 min. After heat-treatment in SDS-PAGE sample buffer containing 10 mM dithiothreitol, the reaction mixture was resolved by SDS-PAGE. As the concentration of heparin increased from 0.04 mg/ml to 3.75 mg/ml, the Coomassie brilliant blue-stained protein band of rCYL at ~7 kDa decreased significantly (a). In the incubation of rCYL with heparan sulfate (HS), a reduction in the rCYL band was evident only when HS was used at 4.0 mg/ml (b). In contrast to heparin and HS, reduction in the rCYL band was not observed in the incubation with SP–Sepharose (c) or chitin (d). In (e), rCYL at 0.5 mg/ml was incubated without ("rCYL") or with heparin at 4 mg/ml ("rCYL + Heparin") or heparan sulfate at 4 mg/ml ("rCYL + HS") at 25°C for 1 h. After SDS-PAGE and silver staining of the gel, protein (indicated by arrowheads) in the samples of "rCYL + Heparin" and "rCYL + HS" but not in the sample of "rCYL" were detected around the boundary between the 4.5% stacking gel and the 18% running gel. In (f), rCYL at 0.5 mg/ml was incubated with heparin at 4 mg/ml ("rCYL + Heparin") or without heparin ("rCYL") at 25°C for 1 h. Absorbance of the sample solution at 280 nm was recorded as "Total", prior to size-exclusion chromatography (molecular weight cut-off approximately 7 kDa) equipped in a spin column. Absorbance at 280 nm of the sample solution that passed through the column was recorded as "Eluted". rCYL that was incubated with heparin was entirely eluted from the column, whereas rCYL alone was absorbed partly in the column. Experiments were performed in triplicate per condition, and the mean values ± SD are shown. Statistically significant differences between the sample were determined using the Student's *t*-test and are indicated as ***$p < 0.001$.
